# Supplementary material for: Soybean-Oil-Based CO2-Switchable Surfactants with Multiple Heads
Source: Molecules. 2021 Jul 18;26(14):4342. doi: 10.3390/molecules26144342 (PMC8305017; doi:10.3390/molecules26144342)
Supplement: Supplementary file 1 [file molecules-26-04342-s001.zip › molecules-1282813-supplementary.pdf]

Supporting Information for

Soybean-Oil-Based CO_2-Switchable Surfactants with Multiple Heads

Huiyu Huang[1], Xiaoling Huang[1], Hongping Quan[2] and Xin Su[1],*

1 Polymer Research Institute, State Key Laboratory of Polymer Materials Engineering, Sichuan University, Chengdu 610065, China

2 Oil & Gas Field Applied Chemistry Key Laboratory of Sichuan Province, School of Chemistry and Chemical Engineering, Southwest Petroleum University, Xindu 610500 China

* Correspondence: xinsu@scu.edu.cn

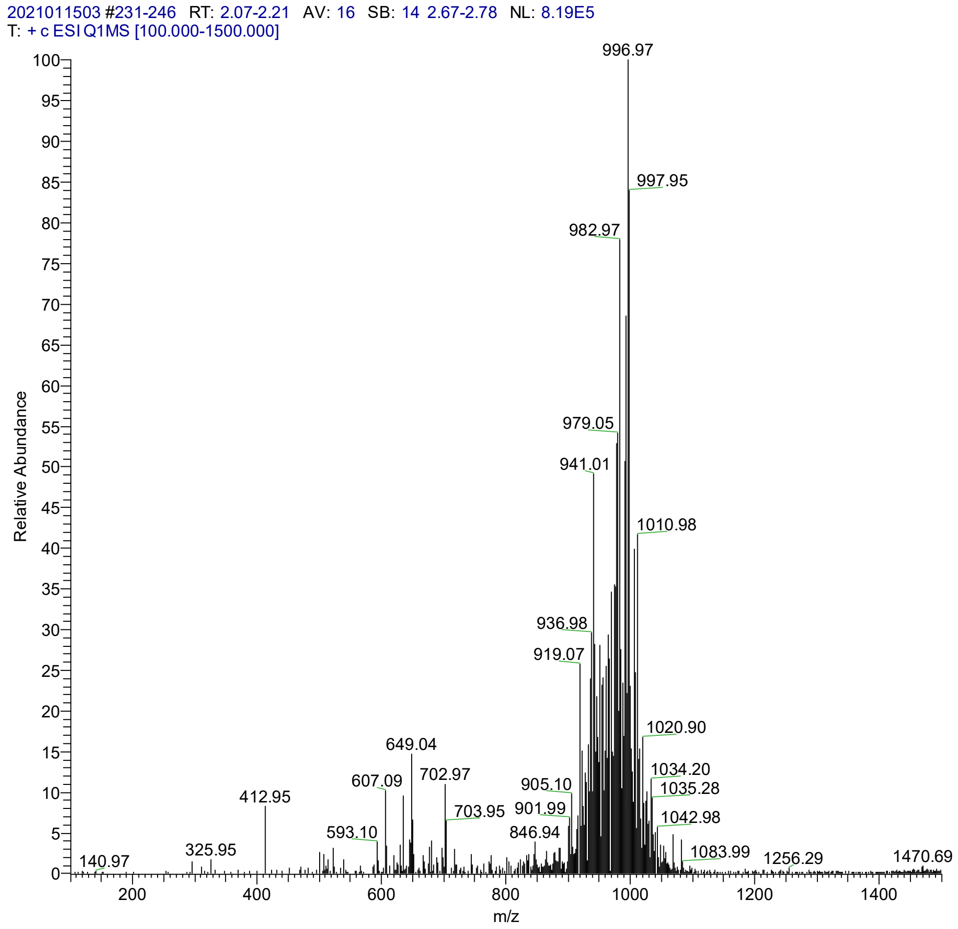

Figure S1. Mass spectrometry result of the oligomeric surfactant

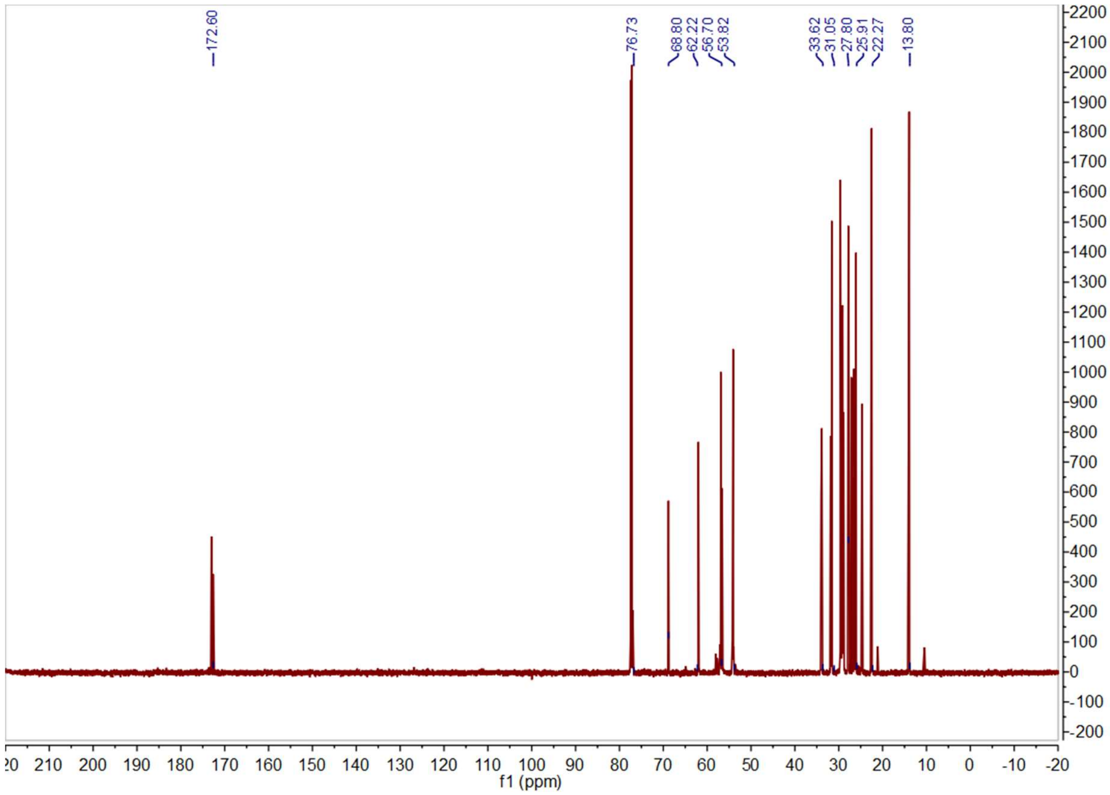

Figure S2. ^{13}C NMR spectrum of the oligomeric surfactant

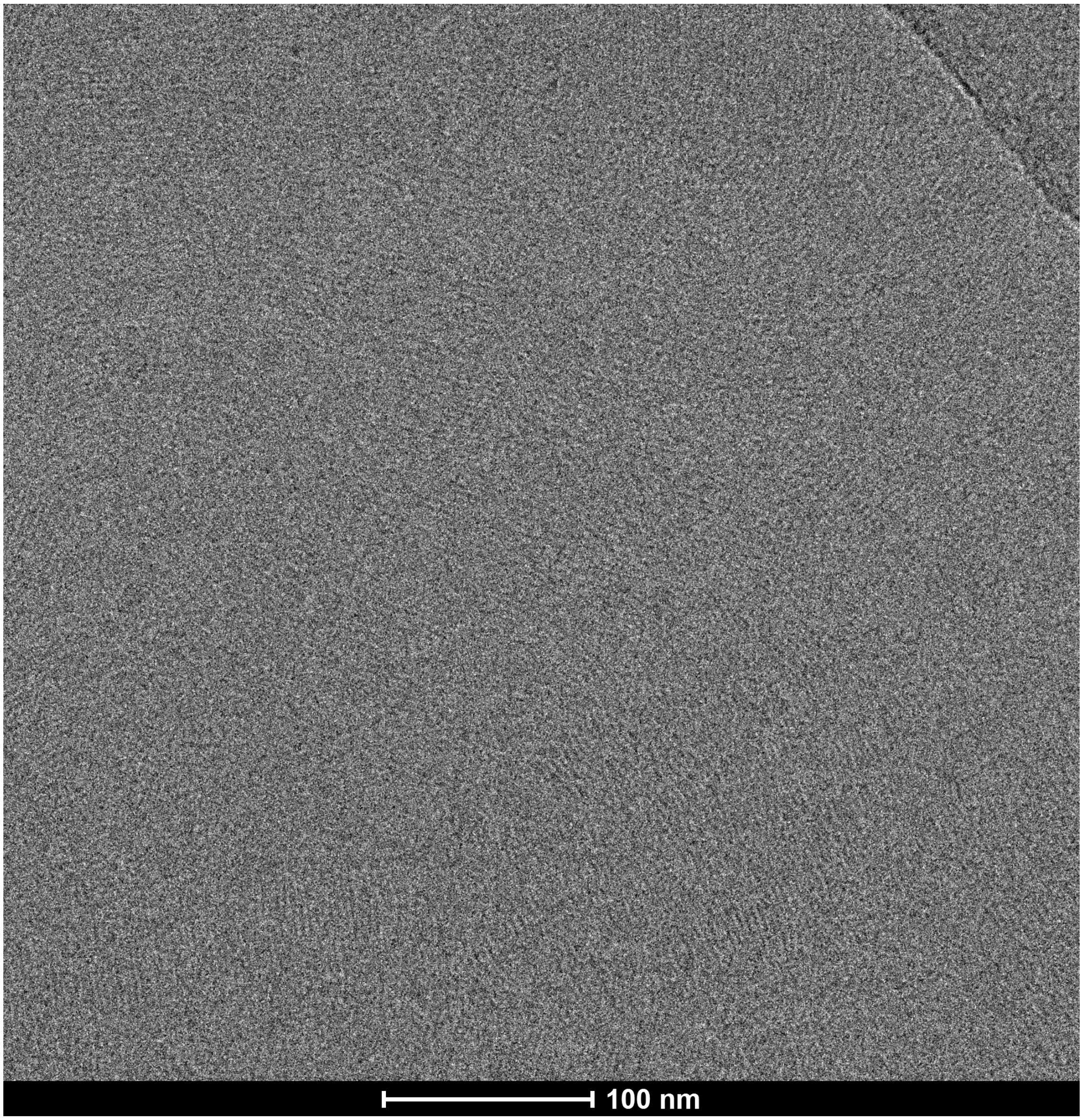

Figure S3. The Cryo-TEM image of aqueous solution for the oligomeric surfactant (12.0 mmol/L) after bubbling CO_2 for 30 min.
